# A series of N-of-1 dietary intervention studies with whole-grain foods and nuts reveals individual predictors of blood pressure and heart rate

**DOI:** 10.1038/s41430-026-01740-3

**Published:** 2026-04-28

**Authors:** Tilly I. T. Potter, Fieke Pepping, Anne J. Wanders, Elizabeth H. Zandstra, Peter L. Zock, Rikard Landberg, Rute Vieira, Baukje de Roos

**Affiliations:** 1 The Rowett Institute, University of Aberdeen, Aberdeen, UK; 2 Unilever Foods Innovation Centre Wageningen, Wageningen, The Netherlands; 3 Division of Human Nutrition and Health, Wageningen University and Research, Wageningen, The Netherlands; 4 Department of Biology and Biological Engineering, Food and Nutrition Science, Chalmers University of Technology, Gothenburg, Sweden; 5 Institute of Applied Health Sciences, University of Aberdeen, Aberdeen, UK

**Keywords:** Biomarkers, Hypertension

## Abstract

**Background/objectives:**

To determine predictors of systolic and diastolic blood pressure (SBP/DBP), of heart rate, and of whole-grains and nuts consumption, on an individual level, using a series of N-of-1 studies.

**Methods:**

Participants were enrolled in individual 24-wk N-of-1 studies, consisting of 8-wk observation, intervention, and follow-up periods. Participants completed personalized morning and evening questionnaires and took their own blood pressure daily. During the intervention period, participants received 3–4 portions of whole-grains and a portion of nuts daily. Fasted blood samples were collected every 4 weeks for analysis of lipids and plasma alkylresorcinol concentrations. Dynamic regression modeling was used to determine factors associated with changes in blood pressure, heart rate, and compliance with the intervention.

**Results:**

12 participants completed their study, with 11 collecting enough data to permit analysis. Dynamic regression modeling identified variables significantly affecting individual blood pressure, heart rate, and consumption of whole-grains and nuts, including sleep quality, weekday, motivation to eat well, alcohol consumption, day of menstrual cycle, outside temperature, and physical activity. Consumption of whole-grains and nuts was associated with a significant (*p* < 0.01) decrease in SBP for one participant, a significant (*p* < 0.05) decrease in DBP for two participants, and a significant (*p* < 0.01) lowering in heart rate for five participants.

**Conclusions:**

The N-of-1 studies uniquely identified individual-level factors that were associated with changes in blood pressure and heart rate, and with compliance to consumption of whole-grains and nuts. N-of-1 studies are imperative to better understand heterogeneity of response and to develop more targeted, personalized and acceptable dietary advice.

**Trial Registration:**

https://clinicaltrials.gov/study/NCT04326686. First registered 20 March 2020.

## Introduction

Diet is an effective treatment for hypertension, one of the most important risk factors for cardiovascular disease [1]. The Dietary Approaches to Stop Hypertension (DASH) diet, focused on increased consumption of fruit and vegetables, whole-grain foods, lean protein-based foods, low-fat dairy, legumes and nuts, and minimizing consumption of red and processed meat and high-fat and added sugar products, has been shown to reduce blood pressure across several studies [2]. Consumption of whole-grain foods may reduce blood pressure, but heterogeneity of response has been observed between and within controlled trials [3, 4]. Studies assessing the effects of whole-grains and nuts on heart rate have also given mixed results [5–7]. Both blood pressure and heart rate are intrinsically variable outcomes —whilst the heterogeneity of response to an intervention may be explained by compliance to whole-grain intake, it may also be affected by several other non-dietary factors that can impact on blood pressure or heart rate, including physical activity levels [8], stress [9], sleep [10], and season or temperature [11].

To accurately determine response at an individual level, particularly to variable outcomes such as blood pressure and heart rate, multiple measurements are required to account for factors such as regression to the mean, measurement error, and fluctuating dietary and non-dietary factors such as compliance, dietary intake, sleep, and stress, which could all affect the outcome [12–14]. This can be done using a series of N-of-1 studies, which are specifically designed to generate statistically robust results using data collected from an individual over time, aiming to draw conclusions on an outcome, like blood pressure, that apply to that person. An interventional N-of-1 study typically takes repeated measurements and/or recordings of both dependent variables (i.e., outcomes) and relevant independent variables, which may affect the outcome(s), in real time, and in the context of day-to-day activities, to determine associations between the independent variables and the outcome(s), on an individual level. Thus, N-of-1 studies can help to better understand associations between dietary interventions and health outcomes for individuals, or individuals that share certain characteristics, more effectively and accurately than standard controlled trials [15, 16].

Modeling Individual Determinants to Intervention using Ecological Tracking (MI-DIET) is a series of N-of-1 studies to determine individual predictors of systolic and diastolic blood pressure (SBP/DBP), of heart rate, and of whole-grains and nuts consumption, in order to assess whether a dietary intervention with whole-grain foods and nuts, or other behavioral, physiological, and environmental factors, affect blood pressure and heart rate, in pre-hypertensive individuals, at an individual level. Compliance with dietary intervention will be assessed through self-reported intake and analysis of plasma alkylresorcinol concentrations, a biomarker of whole-grain wheat and rye [17]. The effect of the dietary intervention on plasma lipids will also be examined.

## Methods

### Setting and ethics

The MI-DIET N-of-1 dietary intervention studies were conducted at the Rowett Institute, University of Aberdeen, United Kingdom, between October 2020 and March 2022. The study was carried out in accordance with the ethical principles of the Declaration of Helsinki, Good Clinical Practice, and complied with the requirements of the Data Protection Act 2018 (applying to the UK’s GDPR standards). Ethical approval was obtained from the Rowett Ethics Panel, University of Aberdeen, United Kingdom. All participants provided written informed consent.

### Eligibility

Participants of any gender aged 18–75 years, with a BMI of 18–35 kg/m^2^ and mildly elevated systolic or diastolic blood pressure levels (SBP 120–140 mmHg and/or DBP 80–90 mmHg), who were low consumers of whole-grain foods ( ≤ 7 portions/week), were recruited through University and NHS websites, social media and small poster adverts. Exclusion criteria were clinical diagnosis of diabetes, hypertension or hypercholesterolemia; unstable or untreated thyroid disorder; taking blood pressure and/or cholesterol-lowering medication; having asthma (owing to coronavirus guidance at the time); following a low-carbohydrate or gluten-free diet; coeliac disease/gluten sensitivity; any food allergies; being on a weight loss diet, or having lost >5 kg in the last six months; any history of an eating disorder; having taken part in another study where whole-grains were provided in the last three months. Participants were compensated for travel costs and their time.

### Study design

At screening, baseline height was measured to the nearest millimeter using a free-standing stadiometer, and baseline weight was measured to the nearest 0.1 kg using calibrated digital scales with the participant wearing light clothing and no shoes. Blood pressure was measured following the protocol proposed by the British Hypertension Society, with a 45 s interval between measurements, until we achieved variability in readings below or equal to 10% or to a maximum of 7 readings. The average of the last three readings was taken to determine eligibility. Variable factors which may associate with blood pressure, heart rate and compliance to the dietary intervention were examined using the following validated questionnaires: the Pittsburgh Sleep Quality Index (PSQI), the Dutch Eating Behavior Questionnaire (DEBQ), the Self-Regulation of Eating Behavior Questionnaire (SREBQ), the UK Short-Form 12 health survey questionnaire (UK SF-12), and the International Physical Activity Questionnaire (IPAQ). A non-validated questionnaire to establish the menstrual cycle and use of contraceptives was also used with female participants. This was followed by a short, semi-structured interview to clarify any responses and determine the times participants would collect their daily measures. Then, a series of individualized interventional N-of-1 studies were conducted, consisting of three periods lasting 8 weeks each in an A1-B-A2 design: an observation period where participants followed their usual diet (A1); a dietary intervention period where whole-grains and nuts were provided (B); and a follow-up period where these foods were no longer provided (A2). The length of the periods was based on the statistical power needed for single-subject N-of-1 dynamic regression modeling ( ~ 50 measurements/study period) [18], and the length of time for any potential blood pressure-lowering to be observed [19]. Data were collected by participants daily throughout the 24-week study in a naturalistic setting. Participants wore a PRO-Diary device (CamNtech Ltd., Cambridge, UK) on the wrist to respond to semi-personalized morning and evening questionnaires, which remained the same throughout each 24-week N-of-1 study. Questionnaires ranged from 5 to 24 questions pertaining to potential predictors that were likely to vary over time, informed by participants’ baseline questionnaires and interviews [16]. The PRO-Diary device also collected tri-axial accelerometry data at 60 s epochs, which was summarized to minutes of vigorous, moderate and light physical activity per day, as well as the number of minutes spent sedentary. Following the British and Irish Hypertension Society (BIHS) guidelines, participants were shown and instructed how to take three consecutive blood pressure and heart rate measurements using a wireless blood pressure monitor (QardioArm, Qardio Inc), around the same time each day, in combination with the Qardio Heart Health smartphone application. Outdoor temperature in Aberdeen (UK) around the time ( ± 2 h) of participants’ blood pressure measurements was recorded to control for temperature effects on blood pressure [11]. For participants for whom a large degree of variation in blood pressure measurement time was identified, the time of measurement was also controlled for. For pre-menopausal female participants, menstrual cycle day was estimated using evening questionnaire responses on menstrual symptoms. From week 2 of each N-of-1 study, participants completed one electronic 24 h dietary intake questionnaire (Intake24, Newcastle University, UK), and every 4 weeks thereafter, to monitor any changes in diet over the course of each study. Every four weeks, starting in week 0, participants arrived at the Human Nutrition Unit at the Rowett Institute, Aberdeen, fasted, for collection of a single 10 ml venous blood sample from the antecubital vein into an EDTA vacutainer to measure blood lipids in plasma or whole blood (with the relevant methodology being directed by COVID-19 measures at the time), and alkylresorcinol concentrations in plasma.

### Whole-grain and nuts intervention

During the 8-wk intervention period (B), participants were provided with their choice of whole-grain breakfast cereals, breads, pasta and snacks (e.g., oatcakes and crispbread) to enable consumption of 3–4 portions of whole-grain foods per day. Most products were whole-grain wheat or rye products, with a smaller number of oat-based products available, as plasma alkylresorcinol concentrations only reflect whole-grain wheat and rye intake [17]. Participants were also provided with a mixture of unsalted nuts: 50% walnuts, 25% almonds and 25% hazelnuts (aside from one participant, who had pecans in place of walnuts), and were recommended to consume one handful per day (approximately 30 g), equivalent to one portion [20]. Participants were provided with a guide to the types of foods recommended to be included and avoided in the DASH diet and were told that they could align their diet more closely with the DASH diet during the intervention if they wished to do so. Participants were recommended to eat the whole-grain foods in place of refined grain products and were given suggestions for how to eat the nuts, e.g., as a snack or cereal topping.

### Laboratory analysis

Lipids were either assessed in fasting whole blood samples using an Alere Cholestech LDX Lipid Profile Cassette in conjunction with an Alere Cholestech LDX Analyzer (Abbott), or analyzed in plasma using a KONE Clinical Analyzer (Thermo Scientific) after spinning at 3500 rpm for 15 min at 4 °C. Plasma was stored at—70 °C for the analysis of plasma alkylresorcinols—the sum of alkylresorcinol homologues C17:0, C19:0, C21:0, C23:0 and C25:0—using liquid chromatography with tandem mass spectrometry on a QTRAP 6500+ (AB SCIEX, Framingham, MA, USA) as described elsewhere [21], to assess its association with self-reported whole-grain intake.

### Data analyses

All analyses were performed using R software (version 4.1.0). Any variables with high amounts of missing data ( > 40%) were not analyzed. For the remaining variables, missing data were imputed using the mean of the previous and subsequent three measurements in the case of numeric data, or using the most frequent response in the case of categorical data. The percentage of actual sleep time in 24 h was calculated by self-reported “light off to sleep” time and “waking up time”, minute-by-minute accelerometry data from the PRO-Diary devices using MotionWare software (CamNtech, Cambridge, UK), or a set of algorithms to calculate sleep length, depending on the data provided by each participant. Minutes spent sedentary and during light, moderate and vigorous physical activity per day were calculated using cutoff points obtained from the PRO-Diary in a previous study [22].

Pearson’s correlation was used to assess the association between self-reported whole-grain food consumption and plasma alkylresorcinol concentrations for each participant separately, and between the average number of portions of whole-grains consumed per day, and percentage of days with self-reported nuts consumption over the preceding 4 weeks, with plasma triglycerides, and total, LDL and HDL cholesterol concentrations.

The variability of all predictors and outcomes over time (by day of the study) was examined visually per individual before their use in dynamic regression modeling, using the package *ggplot2* [23]. Variables were not included in the modeling process where the overall amount of variation was low upon visualisation. The outcomes SBP and DBP were treated as longitudinal numeric data, while portions of whole-grain foods consumed were treated as longitudinal count data, and nuts consumption (Y/N) was treated as longitudinal binary data. Modeling of the outcomes SBP, DBP and heart rate was done using linear dynamic regression, applying the *glm* function within base R. Dynamic Poisson regression was used for modeling portions of whole-grain foods consumed. When assumptions for Poisson regression were not met, negative binomial regression, quasi-Poisson regression, or zero-inflated Poisson regression, as appropriate, were applied using the *MASS* [24] or *pscl* packages [25]. For nuts consumption, Firth’s bias-reduced dynamic logistic regression [26] was applied using the *logistf* package [27].

Dynamic regression models, which are regression models that include lagged variables to control for the effect of time, were used to model each outcome over time, for each participant separately, as described previously elsewhere [16]. Inference is conditional on each individual’s time series and is not intended for population-level generalization. Two sets of covariates, which vary over time, X_t_ and D_t_, were considered. X_t_ describes exogenous variables, such as trend over time and day of the week, endogenous variables such as period of the study (A1, B, A2), and predictor variables taken from the morning and evening questionnaires and actigraphy data. D_t_ are dynamic covariates constructed to summarize the history up to time *t* of responses Y_1_… Y_t_ for the individual, known as “lagged” variables, to adjust for autocorrelation. In the first phase of the analysis, potential predictors were analyzed separately, to identify those predictors that were significantly (*p* < 0.05) associated with each of the individual outcomes (e.g. systolic and diastolic blood pressure, heart rate, (half) portions of whole-grains consumed, and nut consumption (yes/no)), with models being adjusted for time trend (study day number), study period (A1, B, A2), day type (weekday, weekend), and any necessary lagged variables. Subsequently, for the second phase of the analysis, only predictors identified as significant in the first phase of the analysis, including all the exogenous and endogenous variables taking account of time (day number, study period and study duration), were included in a multivariable interrupted time-series model. The best-fitting multivariable model was selected according to Akaike’s Information Criterion; we would assess if a simpler model, where variables that were no longer significant were removed, would result in a lower Akaike Information Criterion (AIC) value, indicating a better-fitting model. We would also explore significant interactions between variables, such as day number and study period, as we would logically expect any reduction in blood pressure during period B to occur gradually over time. Multicollinearity was assessed using variance inflation factors for the predictors included in the final model using the *car* package [28].

## Results

### Descriptive data

**Fig. 1:**
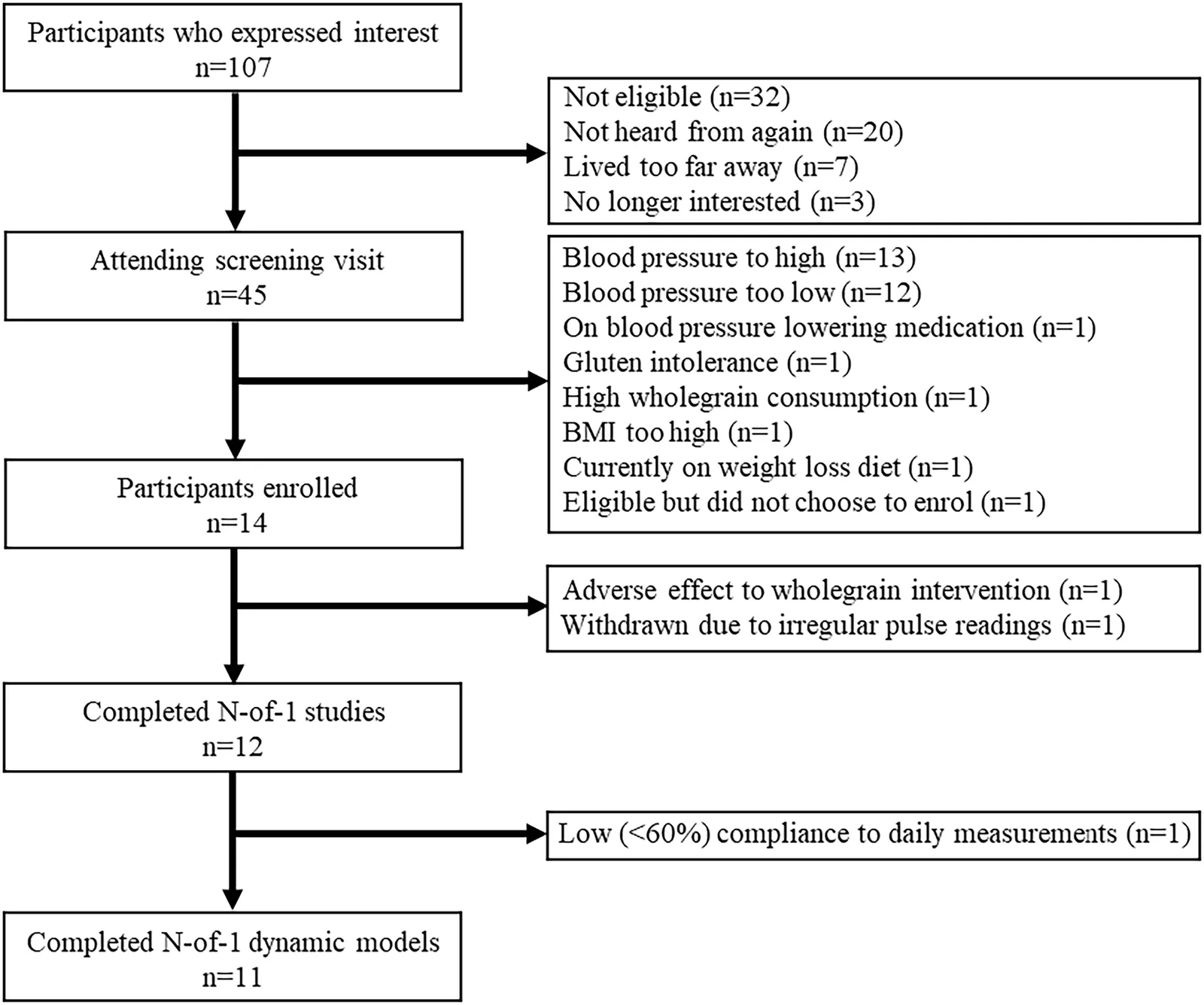
Participant recruitment flow diagram.

107 individuals expressed an interest in taking part, and 45 attended a screening visit. Of these, 15 participants were eligible and 14 enrolled in an N-of-1 study. 12 participants completed their N-of-1 study, with 11 collecting an adequate amount of data to permit analysis of their individual data (Fig. 1). Table 1 summarizes the characteristics of participants who completed their N-of-1 study. Despite recruiting participants with mildly elevated blood pressure levels at screening, three participants had average SBP levels below 120 mmHg throughout their N-of-1 study, while only two participants consistently had DBP levels in the pre-hypertensive range. Most participants were approximately weight stable during the study period, apart from one participant who lost ~20 kg.

**Table 1:** Baseline characteristics of participants who completed their individual N-of-1 study.

	Mean (range) or ratio
Age (years)	44 (29–67)
Sex (female/male)	8/4
BMI (kg/m^2^)	29.0 (24.3–33.8)
Systolic blood pressure (mm Hg)	130 (121–138)
Diastolic blood pressure (mm Hg)	83 (73–89)
Heart rate (BPM^a^)	72 (48–114)
Occupational status (full-time employment/education/retired)	10/2
Total cholesterol (mmol/L)	5.0 (3.3–7.1)
LDL cholesterol (mmol/L)	2.9 (1.4–5.0)
HDL cholesterol (mmol/L)	1.3 (0.5–2.2)
Triglycerides (mmol/L)	1.4 (0.5–4.7)
Self-report days (n)	173 (161–187)
Missing self-report data (%)	12 (3–53^b^)

^a^BPM: beats/min.

^b^One participant had >40% of self-report data missing, meaning that dynamic regression modeling could not be conducted for this participant. Subsequent results tables, therefore, exclude this participant.

Most (8 of 11) participants consumed whole-grain foods less than once per day in the first study period (A1), and all participants increased their consumption in the intervention period (B), although five participants failed to increase their consumption to an average of 3–4 portions per day as recommended. All participants had higher levels of whole-grain consumption in the follow-up (A2) period compared with the start of the study, apart from one participant whose consumption stayed the same. The proportion of days where nuts were consumed was higher in the intervention period (B) compared with period A1 across all participants, and in all but one participant the proportion of days where nuts were consumed was higher in period A2 than period A1 (Table 2 and Supplementary Fig. 1). Using data from the Intake24 diet questionnaires completed every 4 weeks, there appeared to be no change in the proportion of other foods consumed which aligned with a DASH-style dietary pattern (fruit and vegetables, fish, low fat dairy/alternatives, lean protein foods, pulses and unsalted nuts) between study periods (Supplementary Fig. 1). There were also no differences in intakes of macro- or micro-nutrients between study periods (data not shown). There were no significant associations between self-reported whole-grain intake and total alkylresorcinol concentrations. For participant 08, there was a significant negative correlation between average whole-grain consumption and total cholesterol (*R* = –0.88, *p* = 0.02) and LDL cholesterol (*R* = –0.92, *p* = 0.009), and between nuts consumption and total (*R* = –0.9, *p* = 0.016) and LDL cholesterol (*R* = –0.96, *p* = 0.002) (Supplementary Fig. 2).

**Table 2:** Average systolic and diastolic blood pressure, heart rate, portions of whole-grain foods consumed per day, and % nut consumption, for each participant, and during each study period.

	Participant										
Period	01	02	04	05	06	07	08	09	10	11	12
Systolic blood pressure (mmHg)											
A1	142.8 (6.26)	123.9 (4.58)	136.9 (7.60)	116.6 (6.02)	120.4 (6.82)	127.1 (7.07)	119.4 (8.04)	128.6 (7.75)	118.7 (5.74)	126.5 (9.72)	113.5 (5.20)
B	136.3 (7.43)	124.3 (4.04)	128.7 (5.89)	114.5 (5.39)	121.0 (6.87)	120.3 (8.24)	119.0 (6.31)	125.6 (5.61)	122.5 (6.81)	126.8 (7.28)	116.9 (4.29)
A2	134.0 (5.52)	126.8 (6.00)	127.6 (4.54)	111.8 (4.67)	120.7 (7.41)	119.6 (7.56)	115.8 (4.71)	128.3 (6.67)	122.1 (7.70)	126.5 (6.81)	117.5 (5.91)
Diastolic blood pressure (mmHg)											
A1	88.8 (6.05)	77.6 (4.42)	82.7 (8.11)	69.6 (4.67)	65.5 (5.72)	78.6 (4.25)	74.5 (5.90)	76.0 (5.20)	74.8 (4.93)	71.2 (8.95)	72.7 (5.44)
B	82.0 (6.37)	79.3 (3.57)	80.5 (5.62)	66.7 (2.97)	68.5 (6.34)	72.4 (6.83)	74.7 (5.34)	75.0 (5.20)	78.9 (4.71)	68.5 (6.13)	76.7 (3.43)
A2	80.6 (4.68)	80.9 (4.40)	83.4 (2.71)	63.9 (4.65)	67.6 (5.14)	70.4 (5.49)	73.1 (4.20)	75.3 (4.12)	79.5 (5.07)	67.7 (4.24)	77.0 (4.66)
Heart rate (beats per minute)											
A1	92.4 (9.9)	78.2 (4.3)	68.6 (5.7)	64.9 (8.6)	70.6 (4.6)	67.0 (4.3)	61.5 (6.4)	73.6 (4.8)	87.4 (11.4)	64.8 (7.6)	72.4 (9.6)
B	83.1 (10.8)	79.6 (4.6)	67.4 (5.4)	60.0 (5.2)	73.0 (7.6)	63.4 (4.6)	60.7 (4.9)	71.6 (4.3)	89.5 (9.0)	61.5 (5.1)	77.1 (9.1)
A2	79.1 (9.3)	79.1 (5.7)	66.3 (6.5)	63.0 (4.8)	73.2 (6.3)	62.9 (4.2)	58.6 (4.1)	72.2 (5.5)	88.3 (9.4)	60.1 (4.8)	77.8 (9.7)
Number of whole-grain portions consumed/day											
A1	0.2 (0.39)	0.6 (0.67)	0 (0.19)	0 (0.14)	1.9 (0.94)	0.8 (0.93)	0.6 (0.57)	1.3 (1.44)	0.4 (0.62)	1.4 (0.60)	0.4 (0.65)
B	2.8 (1.54)	4.3 (0.98)	2.3 (0.77)	2.8 (0.99)	3.4 (1.34)	3.0 (1.03)	3.7 (1.27)	3.8 (1.84)	1.6 (0.87)	2.8 (0.82)	3.2 (0.56)
A2	0.8 (1.16)	1.8 (0.97)	0.7 (0.69)	1.2 (1.31)	2.7 (1.22)	2.1 (1.02)	3.1 (1.37)	2.4 (1.76)	0.4 (0.65)	2.5 (0.70)	1.5 (1.15)
% days of nut consumption per study period											
A1	11	0	6	0	14	2	2	14	4	4	66
B	98	86	93	86	73	94	89	83	86	89	98
A2	5	25	18	16	43	11	84	74	5	71	77

Data represent mean (SD) from collected values only, e.g., those collected prior to imputation of missing values.

### Dynamic regression models

#### Predictors of SBP and DBP

Table 3 (SBP) and Table 4 (DBP) show the direction of the coefficients and degree of significance, included in the final multivariable dynamic regression models for each participant in each of the columns.

**Table 3:** Predictors associated with systolic blood pressure as revealed by multivariable N-of-1 dynamic linear regression modeling.

		Participant										
		01	02	04	05	06	07	08	09	10	11	12
Time (day of study)		↓	*	***	**	**		↑		↑		↓
Study period	*Period B*	↑	↓	↑		↓	**	↑		↑		↓
	*Period A2*	*	+	++		↓	**	↓		↑		↑
Day number * Period B	*(interaction)*	↓	↑			+						↑
Day number * Period A2	*(interaction)*	↑	↑			++						↑
Whole-grain portions	*Lag 0*	++						↓				
Nuts consumption	*Yes (1 portion)*	↓										
Day type	*Weekend (vs weekday)*				↑			↑		+		
Work	*Day off (Lag 0)*		↑					↓				↑
	*Day off (Lag 1)*							+				
Temperature at BP measurement			↑				↓					
BP measurement time		↓									**	
Light off to sleep time	*Lag 0*	↑	↑									
	*Lag 1*		*									
Measured sleep duration	*Lag 0*					↑						
	*Lag 1*					+						
Sleep efficiency					+							
Sleep quality (subjective)	*Lag 0*				↑	**				**		
	*Lag 1*					↑						
Times woken			+									
Subjective times woken up	*Lag 0*						↓		**			
	*Lag 1*						↓					
	*Lag 2*						**					
Busyness		↓	↑									↓
Tiredness			**									
Degree of anxiety									+			
Degree of food restriction					↓	↓		↓				
	*Lag 1*					↓						
Motivation to eat well												*
Location	*Outdoors*				↓		↓					
	Other indoors*				↓		↓					
	Travelling*				↓		↓					
	*At home*	*										
	*Holiday destination*											
	Other*	↓										
	*Outdoors -Lag 1*				↓							
	Other indoors * -Lag 1				↓							
	Travelling* -Lag 1				↑							
	*At home -Lag 1*	↑										
	*Holiday destination -Lag 1*											
	Other * -Lag 1	↑										
Eating out	*Yes, once*		↑									
	*Yes, more than once*		↑									
Portions pastry/d	*Lag 0*			*								
	*Lag 1*			+								
Portions processed meat/d					↑							
Portions of cheese/d	*Lag 0*					↑						
	*Lag 1*					↑						
	*Lag 2*					+++						
Portions of chocolate/d							*					
Units alcohol/d	*Lag 0*					↓						
	*Lag 1*					↑						
Menstrual cycle day			++									
Health limiting activities	*Lag 0*					↓						
	*Lag 1*					↓						
	*Lag 2*					↓						
	*Lag 3*					++						
Physical activity, minutes	*Light PA -Lag 0*		↑		↑				↓			
	*Light PA -Lag 1*								++			
	*Moderate PA -Lag 0*	↓			↑	↑						
	*Moderate PA -Lag 1*	↓			↓	↓						
Rugby practice	*Yes*			*								
Weight training	*Yes*			↑								
Cycling	*Yes*										*	
Childcare	*Yes -Lag 0*										↓	
	*Yes -Lag 1*										↑	
	*Yes -Lag 2*										↓	
Family influence activity												↑
Systolic BP	*Lag 1*		↑		↑	↑	+		↓	↑	++	+
	*Lag 2*		↓		++		↑		++			+
	*Lag 3*		↓				++		++			+

↑: positive but non-significant coefficient.

↓: negative but non-significant coefficient.

*: significant negative coefficients.

+: significant positive coefficients.

*BP* blood pressure, *PA* physical activity.

*/+ = *p* < 0.05.

**/++ = *p* < 0.01.

***/+++ = *p* < 0.001.

Lag X corresponds to the effect of the variable on day X, where X = 0 represents the present day, X = 1 represents the previous day, X = 2 represents two days before, etc.

Only predictors identified as significant in the first phase of the analysis were included in the multivariable models for each individual.

**Table 4:** Predictors associated with diastolic blood pressure (DPB) as revealed by multivariable N-of-1 dynamic linear regression modeling. Only predictors identified as significant in the first phase of the analysis were included in the multivariable models for each individual.

		Participant										
		01	02	04	05	06	07	08	09	10	11	12
Time (day of study)		↓	**	**	↓		↓	*		+	***	↑
Study period	*Period B*	↑	↓	↑	↓	↑	+	↓		↑	↓	↑
	*Period A2*	*	↑	++	↓	↑	↓	↓		↓	↓	↑
Time * Period B	*(interaction)*	*	++				*	+			++	
Time * Period A2	*(interaction)*	↑	↑				+	+			*	
Whole-grain portions						↓						
Nuts consumption	*Yes (1 portion)*	↓										
Day type	*Weekend (vs weekday)*				↑			↑		↑	↓	↓
Work	*Day off -Lag 0*		+			↑		↓				↑
	*Day off -Lag 1*							++				
Temperature at BP measurement			↑					+++				
BP measurement time		*									↓	
Light off to sleep time	*Lag 0*	+							+			↑
	*Lag 1*											+
Measured sleep duration	*Lag 0*					↑						+
	*Lag 1*					↑						
Sleep efficiency					++							
Subjective sleep quality						↓						
Sleep disturbed-being too hot												↑
Subjective times woken up	*Lag 0*						↑		*			
	*Lag 1*						↓					
	*Lag 2*						*					
Location	Outdoors* -Lag 0								↓	↑		
	At home* -Lag 0								↑	↑		
	*Other indoors -Lag 0*								↑			
	*Travelling -Lag 0*								+			
	*Holiday destination -Lag 0*								↑			
Well-restedness		+				↓						
Busyness			↑			↓			↓	↓		↓
Tiredness	*Lag 0*		*									↓
	*Lag 1*											↑
Less interest in activities			↓									
Degree of boredom										*		
Motivation to eat well	*Lag 0*					↑		+				**
	*Lag 1*					↑						
	*Lag 2*					↓						
Degree of food cravings								*				
Degree of food restriction								↓		↓		
Eating out	*Yes*											*
Portions of chocolate/d	*Lag 0*					↑	*					
	*Lag 1*					↓						
Units of alcohol/d	*Lag 0*					↓						
	*Lag 1*					↑				↓		
Physical activity - mins	*Sedentary*		↑									
	*Light PA -Lag 0*		↑						↑			
	*Light PA -Lag 1*								++			
	*Moderate PA -Lag 0*				↓	↑		↓				
	*Moderate PA -Lag 1*				↓	↓						
	*Moderate PA -Lag 2*					↓						
Weight training	*Yes*			+								
Cycling	*Yes*										*	
Childcare	*Yes -Lag 0*										↓	
	*Yes -Lag 1*										↑	
	*Yes -Lag 2*										↓	
Diastolic blood pressure	*Lag 1*		↓		↓	+	↑	↑			↑	+++***
	*Lag 2*		↑		+	↑	↑	↑				↑
	*Lag 3*		*			↑		↑				+
	*Lag 4*											↑

*BP* blood pressure, *PA* physical activity.

↑: positive but non-significant coefficient.

↓: negative but non-significant coefficient.

*: significant negative coefficients.

+: significant positive coefficients.

*/+ = *p* < 0.05.

**/++ = *p* < 0.01.

***/+++ = *p* < 0.001.

Lag X corresponds to the effect of the variable on day X, where X = 0 represents the present day, X = 1 represents the previous day, X = 2 represents two days before, etc.

In terms of overall time trends and time trends at period level, 4 participants (02, 04, 05 and 06) had significant reductions in SBP over the course of the entire study (*p* < 0.05, *p* < 0.001, *p* < 0.01, and *p* < 0.01, respectively), whereas 5 participants (02, 04, 08, 10, and 11) had significant changes in DBP over the course of the whole study; for 4 of these participants (02, 04, 08, and 11) DBP significantly reduced over time (*p* < 0.01, *p* < 0.01, *p* < 0.05, and *p* < 0.001, respectively).

In terms of main effects in periods B and A2, compared with period A1, accounting for any time-trends unrelated to the interventions, average SBP was significantly lower in participant 07 in periods B and A2 (both *p* < 0.01), and significantly lower in participant 01 in period A2 (*p* < 0.01). Furthermore, average DBP was significantly lower in participants 01 and 07 in period B (*p* < 0.01). Other significant predictors that explained changes in blood pressure over time included blood pressure measurement time, outside temperature, tiredness, motivation to eat well, menstrual cycle day, physical activity, and sleep quality. For example, for participant 10, weekend days were associated with significantly higher SBP values compared with weekdays (*p* < 0.05), and for participant 02, days off from work were associated with significantly higher DBP values compared with workdays (*p* < 0.05). For participant 09, more minutes of light physical activity the day before were associated with significantly higher SBP and DBP values the following day (both *p* < 0.01). For participants 06 and 10, a better self-reported sleep quality the night before was associated with significantly lower SBP values (both *p* < 0.05), whereas for participants 01 and 09, a later sleep time the previous night was associated with significantly higher DBP values (both *p* < 0.05). For participant 02, significantly higher SBP values were recorded later in their menstrual cycle compared with earlier in their cycle (*p* < 0.01).

#### Predictors of heart rate

Table 5 shows the direction of the coefficients and degree of significance of all predictors that were significantly associated with heart rate in the final dynamic regression models for each participant, in each of the columns. Average heart rate was significantly lower in four participants (01, 06, 08 and 11) in period B compared with period A1 (*p* < 0.01, *p* < 0.001, *p* < 0.001 and *p* < 0.01, respectively), whereas average heart rate was also significantly lower for participant 01 in period A2 (*p* < 0.01). The average heart rate was significantly lower in one participant (04) due to nut consumption (*p* < 0.05). Other significant predictors of daily heart rate values included weekday (weekend versus weekdays), measurement time, outside temperature, menstrual cycle day, sleep quality, degree of tiredness, alcohol consumption, social factors and physical activity levels. For example, for participants 06, 07, and 08, a higher outside temperature was associated with significantly higher heart rate readings; this was only the case for participant 06 during period B. For participants 02 and 08, being at a later phase in their menstrual cycle was associated with significantly higher heart rate readings (*p* < 0.001 and *p* < 0.05, respectively). For participants 01, 08, and 12, a later measurement time was associated with significantly lower heart rate readings (*p* < 0.01, *p* < 0.001, *p* < 0.05, respectively). For participants 02, 06, 09 and 12, alcohol consumption was associated with a significantly higher heart rate (*p* < 0.05, *p* < 0.05, *p* < 0.01, *p* < 0.001, respectively). For participants 01, 05 and 09, a higher amount of light or moderate physical activity was associated with a significantly higher heart rate the same day (*p* < 0.01, *p* < 0.05, *p* < 0.05, respectively).

**Table 5:** Predictors associated with heart rate as revealed by multivariable N-of-1 dynamic linear regression modeling. Only predictors identified as significant in the first phase of the analysis were included in the multivariable models for each individual.

		01	02	04	05	06	07	08	09	10	11	12
Time (day of study)		**+**		**↑**	*	**++**	**↑**	***	**↑**	**↓**	**	**↑**
Study period	*Period B*	**	**↑**	**↓**	**↓**	***	**+**	***	**↓**	**↑**	**	**↑**
	*Period A2*	**	**↑**	*		**↓**	**↓**	**↑**	**↓**	**↑**	**↑**	**↑**
Day number * Period B	*(interaction)*			**↑**			**	**+++**			**++**	
Day number * Period A2	*(interaction)*			*	*		**↓**				**↑**	
Whole-grains portions	*Lag 0*		**									
	*Lag 1*											
Whole-grain portions * Period B	*(interaction)*		**+**									
Whole-grain portions * Period A2	*(interaction)*		**+**									
Nuts consumption	*Yes (1 portion)*			*								
Day type	*Weekend*									***	**++**	
Work	*Day off –Lag 0*								*			
	*Day off -Lag 1*									**++**		
Temperature at BP measurement						**↓**	**+++**	**++**				
Temperature * Period B	*(interaction)*					**++**						
Temperature * Period A2	*(interaction)*					**↑**						
Light off to sleep time	*Lag 0*											
	*Lag 1*											**+**
BP measurement time		**							***			*
Well-restedness	*Lag 0* *L*											
	*Lag 1*											
	*Lag 2*	*										
Subjective sleep quality	*Lag 0*											
	*Lag 1*							*				
Subjective times woken up							**++**					
Busyness	*Lag 0*											**
	*Lag 1*					**						
Tiredness	*Lag 0*					**						
	*Lag 1*											**+++**
Anxiousness		**+**										
Sadness	*Lag 0*											
	*Lag 1*							**				
Menstrual cycle			**+++**					**+**				
Social setting	*Yes, once*	**+++**							**+**			
Bread slices/d											**+**	
Units of alcohol/d			**+**			**+**			**++**			***
Physical activity - mins	*Light PA*	**++**										
	*Moderate PA -Lag 0*				**+**				**+**			
	*Moderate PA -Lag 1*				**↓**							
Running	*Yes*				**+++**							
Rugby practice				**+++**								
Rugby practice * period B	*(interaction)*			**								
Rugby practice * period A2	*(interaction)*			***								
To the gym	*Yes -Lag 0*											
	*Yes -Lag 1*									**+**		
Heart rate	*Lag 1*	**+**		**↑**	**+**							**↑**
	*Lag 2*			**↓**	**↑**							**+**
	*Lag 3*			↑	↑							

*BP* blood pressure, *PA* physical activity.

↑: positive but non-significant coefficient.

↓: negative but non-significant coefficient.

*: significant negative coefficients.

+: significant positive coefficients.

*/+ = *p* < 0.05.

**/++ = *p* < 0.01.

***/+++ = *p* < 0.001.

Lag X corresponds to the effect of the variable on day X, where X = 0 represents the present day, X = 1 represents the previous day, X = 2 represents two days before, etc.

#### Predictors of whole-grain and nuts consumption

Table 6 (whole-grains) and Table 7 (nuts) show the direction of the coefficients and degree of significance of all predictors that were significantly associated with whole-grain and nuts consumption, per participant, in each of the columns. Whole-grain consumption could not be modeled for participant 05, while nut consumption could not be modeled for participant 12 due to constraints in variability on the available data. Seven participants (02, 04, 06, 07, 08, 10, and 12) consumed significantly more portions of whole-grains in period B, whereas six participants (02, 04, 06, 07, 08, and 09) consumed significantly more portions of whole-grains in period A2, compared with A1 (all *p* < 0.05), showing that participants largely maintained the habit of consuming whole-grains. Other significant predictors of whole-grain consumption included weekday, degree of calmness or anxiety, and where participants spent most of the day. For example, for participants 07 and 08, weekend days and days off work, respectively, were associated with significantly lower consumption of whole-grains compared with weekdays and work days (*p* < 0.05). For participant 01, reporting feeling calmer was associated with consuming more portions of whole-grains (*p* < 0.01), whereas for participant 09, a higher level of anxiety was associated with significantly lower consumption of whole-grains (*p* < 0.05). For participant 10, being at home was associated with significantly higher consumption of whole-grains compared to being at work (*p* < 0.05). For seven participants (01, 02, 04, 05, 07, 08, 10), consumption of nuts was more likely in period B (all *p* < 0.01), whereas for six participants (02, 04, 05, 08, 09, 10), consumption of nuts was more likely in period A2 (all *p* < 0.05), compared with period A1. Other significant predictors of nuts consumption included weekday, location where participants spent most of their day, degree of anxiety, and degree of food restriction. For example, for participants 01 and 08, weekends were associated with a greater and lower likelihood of nuts consumption, respectively (both *p* < 0.05). For participants 06 and 11, a higher motivation to eat well was associated with a significantly greater likelihood of nuts consumption (*p* < 0.001, *p* < 0.05. respectively), and for participants 08 and 11, a higher degree of food restriction was also associated with a significantly higher likelihood of nuts consumption (*p* < 0.05 and *p* < 0.001, respectively). For participants 08 and 09, higher anxiety was associated with a significantly greater and lower likelihood of nuts, respectively (both *p* < 0.01).

**Table 6:** Predictors associated with (half) portions of whole-grains consumed per day, using multivariable N-of-1 Poisson regression, negative binomial regression, quasipoisson regression or zero-inflation Poisson regression modeling. Only predictors identified as significant in the first phase of the analysis were included in the multivariable models for each individual.

		Participant										
		01	02	04	05	06	07	08	09	10	11	12
Time (day of study)		**	**↓**	*		**+**	**↑**		**↑**	**↓**	**↓**	**↑**
Study period	*Period B*	**↑**	**+++**	**+++**		**++**	**+++**	**+++**	**↑**	**++**	**↓**	**+**
	*Period A2*	**↑**	**+++**	**+++**		**+**	**+++**	**+++**	**+++**	**↑**	**↑**	**↓**
Day number * Period B	*(interaction)*	**++**				**↓**			**↓**		**++**	
Day number * Period A2	*(interaction)*	**++**				*			**↓**		**↑**	
Day type	*Weekend (vs weekday)*	**↓**				**↓**	*	**↑**	**↓**	**↓**	**↓**	
Work	*Day off*							*				
Sleep efficiency	*Lag 0*		**↑**									
	*Lag 1*		**+**									
Whole-grain consumption	*Lag 1*	**↑**	**++**	**↓**		**↑**		**↓**	**↑**	**↑**	**↑**	**↑**
	*Lag 2*					**↑**				**↑**	**↑**	
Whole-grain enjoyment									**↑**			
Sleep quality	*Lag 0*	**↓**										
	*Lag 1*	**↓**										
	*Lag 2*	**↑**										
Number of waking bouts							**++**					
Sleep disturbed- being too hot									**↑**			
Morning calmness	*Lag 0*	**++**										
	*Lag 2*											
Location	At home*	**↑**				**↓**			**↓**	**+**		
	*Other indoors*					**↓**			**↓**			
	*Travelling*					**↓**			**↓**			
	Outdoors*					**↓**			**↓**	**↓**	**↑**	
	*Holiday destination*					**↑**			*			
	Other*	**+**									**++**	
	*At home –Lag 1*	**↓**										
	Other* -Lag 1	*										
Social setting	*Yes, once*								↓			
	*Yes, > once*								↓			
Eating out	*Yes, once (Lag 0)*								↑			
	*Yes, > once (Lag 0)*								↓			
	*Yes, once (Lag 1)*								↑			
	*Yes, > once (Lag 1)*								↓			
	*Yes, once (Lag 2)*								↓			
	*Yes, > once (Lag 2)*								↓			
Busy	*Lag 0*					↑				↑		↑
	*Lag 1*											↓
Evening tiredness	*Lag 0*					↑						↑
	*Lag 1*											↓
	*Lag 2*											↓
Evening sadness						**++**						
Degree of anxiety									*			
Degree of boredom										**↑**		
Degree of food restriction	*Lag 0*					**↑**	***	**↑**		**↑**		
	*Lag 1*							**+**		**↑**		
Degree health limits activities						**↑**						
Units of alcohol per day						**↓**		*				
Chocolate portions	*Lag 0*	**↓**							**↑**			
	*Lag 1*	**↑**							**↓**			
	*Lag 2*	**↑**										
Bread slices											**+++**	
Light physical activity												**↑**
Moderate physical activity	*Lag 0*										**↑**	
	*Lag 1*										**+**	
*Exp ZIP zero inflation model*												
Time (day of study)		↓								↓		*
Study period	*Period B*	↓								↓		↓
	*Period A2*	↓								↑		↑
Day number * Period B	*(interaction)*	↑										
Day number * Period A2	*(interaction)*	↑										
Day type	*Weekend (vs weekday)*	**+**								↑		
Work	*Day off*											
Whole-grain consumption	*Lag 1*	**↓**								*		**
	*Lag 2*									**↓**		
Location	At home*	**↑**								**↓**		
	Outdoors*									**↓**		
	Other*	**↑**										
	*At home -Lag 1*	*										
	Other* -Lag 1	↓										
Sleep quality	*Lag 0*	↓										
	*Lag 1*	↑										
	*Lag 2*	↓										
Morning calmness		↓										
Busyness	*Lag 0*									**		↑
	*Lag 1*											↓
Tiredness	*Lag 0*											↓
	*Lag 1*											↓
	*Lag 2*											↑
Degree of boredom										↑		
Degree of food restriction	*Lag 0*									**↓**		
	*Lag 1*									**+**		
Chocolate portions	*Lag 0*	**↓**										
	*Lag 1*	**+**										
	*Lag 2*	**↑**										
Light PA in minutes												**↑**

*PA* physical activity.

↑: positive but non-significant exponentiated coefficient.

↓: negative but non-significant exponentiated coefficient.

*: significant exponentiated coefficients less than 1.

+: significant exponentiated coefficients greater than 1.

*/+ = *p* < 0.05.

**/++ = *p* < 0.01.

***/+++ = *p* < 0.001.

Lag X corresponds to the effect of the variable on day X, where X = 0 represents the present day, X = 1 represents the previous day, X = 2 represents two days before, etc. Exponentiated zero-inflation count model (Poisson): participants 01, 10 and 12; exponentiated coefficients model (Poisson): participants 02, 04, 06; exponentiated coefficients model (negative binomial): participants 07, 08, 09; exponentiated coefficients model (quasipoisson): participant 11.

**Table 7:** Predictors associated with nuts consumption (Y/N) using multivariable Firth’s bias-reduced dynamic logistic regression. Only predictors identified as significant in the first phase of the analysis were included in the multivariable models for each individual.

		Participant									
		01	02	04	05	06	07	08	09	10	11
Time (day of study)		**↓**	**↑**	**↓**	**↓**		**↑**	**↑**	**↑**	**+**	*
Study period	*Period B*	**+++**	**++**	**+++**	**+++_**		**++**	**+++**	**↓**	**+++**	**↑**
	*Period A2*	**↑**	**+++**	**+**	**+**		**↓**	**+**	**+++**	**+**	**↓**
Day number * Period B	*(interaction)*		**↓**				**↓**		**↑**	**	**+**
Day number * Period A2	*(interaction)*		*				**↓**		**	**	**++**
Day type	*Weekend (vs weekday)*	**+**						*		**↓**	
Work	*Day off*		**↓**			**↑**					
Light off to sleep time		*									
Measured sleep duration	*Lag 0*					**↓**					**↑**
	*Lag 1*										**
Sleep quality (subjective)	*Lag 0*		**↓**								
	*Lag 1*		**↑**								
	*Lag 2*		**+**								
Well-restedness	*Lag 0*							**↑**			
	*Lag 1*							**↑**			
Nuts consumption	*Lag 1*					**+++**		*	**↑**		**↑**
Motivation to eat well						**+++**					**+**
Morning happiness	*Lag 0*									**↑**	
	*Lag 1*									**↓**	
Location	*Outdoors*				**++**				**↓**		
	At home*				**↓**				**↑**		
	*Other indoors*								**↓**		
	Travelling*				↓				↓		
	*Holiday destination*								↑		
Eating out	*Yes, once*								↓		
	*Yes, more than once*								*		
Busyness	*Lag 0*					**↓**					
	*Lag 1*					**++**					
Degree of boredom							**				
Degree of anxiety	*Lag 0*							**++**	**		
	*Lag 1*							**↓**			
Degree of food restriction	*Lag 0*					**↑**		**+**			**+++**
	*Lag 1*					**↓**					
	*Lag2*					**_++**					
Cheese portions per day						**↓**					
Chocolate portions per day									**↓**		
Units of alcohol per day	*Lag 0*					**↓**					
	*Lag 1*					↓					
Less interest in activities			**								
Physical activity in mins	*Vigorous PA -Lag 0*					**+**					
	*Moderate PA -Lag 0*					*					
	*Moderate PA -Lag 1*					**↑**					
Gym attendance	*Yes - Lag 0*						**+**			**↓**	
	*Yes - Lag 1*									*	

*PA* physical activity.

↑: positive but non-significant coefficient.

↓: negative but non-significant coefficient.

*: significant negative coefficients.

+: significant positive coefficients.

*/+ = *p* < 0.05,

**/++ = *p* < 0.01.

***/+++ = *p* < 0.001.

Lag X corresponds to the effect of the variable on day X, where X = 0 represents the present day, X = 1 represents the previous day, X = 2 represents two days before, etc.

## Discussion

The MI-DIET series of N-of-1 studies is, to our knowledge, the first to assess individual predictors of compliance to a dietary intervention and subsequent physiological response. The series of N-of-1 studies assessed whether consumption of whole-grains and nuts, or other behavioral, psychological, and environmental factors, affects blood pressure and heart rate, which are major risk factors for cardiovascular disease [29], at the individual level. We found that a dietary intervention with whole-grain foods and nuts led to significant reductions in SBP in one participant, significant reductions in DBP in two participants, and significant reductions in heart rate in five participants. N-of-1 dynamic regression models also identified other factors that were associated with individual variation in blood pressure and heart rate, including sleep quality, weekday, outdoor temperature, alcohol consumption, phase of menstrual cycle and minutes of physical activity, amongst others. Predictors of whole-grain and nuts consumption included weekday, where participants spent most of their day, how calm or anxious they felt and the degree to which they restricted their food consumption, for some, but not for others.

Consumption of both whole-grain foods and nuts has been associated with a reduced risk of hypertension in prospective cohort studies [30]. Whole-grain foods, nuts, and fruits and vegetables, all important components of the DASH diet [31], are rich in dietary fiber, which significantly reduced both SBP and DBP in a meta-analysis of randomized controlled trials of 8 weeks or more [32]. Fiber supplementation is often associated with concomitant increases in potassium and magnesium intake, which may provide further blood pressure-lowering benefits [33]. Blood pressure lowering by whole-grain foods and nuts is likely mediated by several mechanisms, including improvements in endothelial function, attenuated insulin response and improvements in mineral absorption [33, 34]. However, on an individual level, blood pressure and heart rate vary considerably over time, due to diet and other lifestyle and environmental factors [8–11], as already acknowledged in the context of another N-of-1 trial [35], and observed in the current series of N-of-1 studies. Co-varying factors are typically not measured and hence controlled for in randomized controlled trials. Many of the co-varying factors in our study have been mechanistically linked to blood pressure and heart rate in previous studies. For example, lack of sleep or poor sleep quality is known to increase hypertension risk [10], whilst regular physical activity is known to reduce blood pressure, and in the short-term may help to lower stress, concomitantly lowering blood pressure [8, 36]. Alcohol has been associated with increased heart rate within 6 h of consumption [37], while factors such as time of day, menstrual cycle phase and outside temperature have also been associated with changes in heart rate [38–40].

N-of-1 dynamic regression modeling revealed significant predictors of individual blood pressure and heart rate trajectories, and of whole-grain and nuts consumption, at an individual level (Tables 3–7). This series of N-of-1 studies was not powered to identify how many portions of whole-grains (or nuts) were required to observe a change in blood pressure. For some participants, reductions in blood pressure were observed over the entire course of the study, which suggests effects other than the dietary intervention led to blood pressure reductions, such as participating in a trial and becoming more health-conscious. Indeed, it is not uncommon to observe changes in behavior and/or improvements in weight when enrolling in an intervention study, even without an active intervention [41]. For example, participant 05 lost a considerable amount of weight during the study, which is likely to have been the main reason for their systolic blood pressure reduction [42]. This highlights the importance of including an observation period, as we did in this study, to determine whether changes over time could be attributed to the dietary intervention.

In this series of N-of-1 studies, consumption of whole-grains and nuts was higher or more likely in the follow-up period (A2) of the study compared with the observation period (A1), despite the products no longer being provided after the intervention period (B). This suggests participants formed a habit of consuming these foods over the course of the study [43]. However, participants did not appear to align their diets more closely with the DASH diet aside from their consumption of whole-grains and nuts, suggesting that consuming the products provided was more achievable than making wider changes to their diets. Five of the 11 participants failed to integrate or maintain an average of three or more portions of whole-grains per day during the 8-week intervention period, which may have affected plasma alkylresorcinol concentrations, which are a marker for whole-grain wheat and rye consumption [17]. Overall, alkylresorcinol concentrations only weakly correlated with the daily whole-grains self-report data; we hypothesize that alkylresorcinol concentrations may have been affected by the use of fasted plasma samples, due to the relatively short ( ~ 4 h) half-life of alkylresorcinol concentrations [44], or by the fact that whole grains were not solely provided by wheat and rye.

A key strength of this series of N-of-1 studies is that all variables used for dynamic regression modeling were measured continuously, facilitating the identification of different significant predictors of physiological response and compliance at an individual level. Such a level of detail could not be achieved using a traditional, group-based study design using a single set of measurements prior to and after the dietary intervention to measure change in blood pressure. Controlling for time and its interaction with the different study periods enabled us to identify whether significant changes in blood pressure or heart rate occurred, and if these could be attributed to the dietary intervention. A limitation of this study was the use of a single set of baseline blood pressure measurements and a whole-grains consumption questionnaire to establish eligibility for the study at screening. Three participants consistently had at-home blood pressure measurements in the healthy range, and three participants consumed >1 portion of whole-grains/d in the first observation period. This led to a lower likelihood of observing blood pressure-lowering effects. Secondly, while efforts were made to identify relevant variable factors for each participant that may have been associated with the outcomes of interest, the types of questions asked each day were limited by the information gathered from the baseline questionnaires and the pre-study interview. Some data could not be used due to a lack of variation in response to certain questions.

In conclusion, this series of N-of-1 studies could identify individual participants with significant reductions in blood pressure or heart rate over the course of the study, and whether these reductions could be attributed to a dietary intervention with whole-grain foods and nuts. The MI-DIET studies also identified other behavioral, physiological and environmental factors that significantly affected blood pressure and heart rate at an individual level. The high compliance to daily blood pressure monitoring and questionnaires over 24 weeks ( > 90%) showed that this type of study design, along with analysis using dynamic regression modeling, offers significant scope for future observational and interventional N-of-1 nutrition studies [15]. For variable outcomes such as blood pressure and heart rate, an N-of-1 approach offers the unique opportunity to follow individuals over time and to reveal the efficacy of dietary interventions on an individual level. This series of N-of-1 studies provides a greater understanding of why people respond differently to similar dietary interventions, and highlights the potential of targeted and personalized approaches to improve compliance with, and the efficacy of, dietary and other lifestyle interventions.

## Supplementary information

Supplementary Data

## Data Availability

Anonymized data described in the manuscript will be made available upon request, pending application and approval by the corresponding author.
