# Supplementary material for: A series of N-of-1 dietary intervention studies with whole-grain foods and nuts reveals individual predictors of blood pressure and heart rate
Source: Eur J Clin Nutr. 2026 Apr 28;80(8):799–813. doi: 10.1038/s41430-026-01740-3 (PMC13493315; doi:10.1038/s41430-026-01740-3)
Supplement: Supplementary file 1 [file 41430_2026_1740_MOESM1_ESM.docx]

**Supplementary Data**

**Supplementary Figure 1.** Average consumption of whole-grain foods, nuts, and the proportion of other foods which aligned with a DASH-style dietary pattern (fruit and vegetables, fish, low fat dairy/alternatives, lean protein-based foods, pulses and unsalted nuts) in study periods A1, B and A2, across all participants. Data were calculated from Intake24 dietary assessment records.

**Supplementary Figure 2**. Body weight, plasma concentrations of total alkylresorcinols, total, LDL and HDL cholesterol, and triglycerides, for the 11 participants, obtained every four weeks during the 24-wk study period.
